# Beta-Hydroxybutyrate Levels and Risk of Diabetic Ketoacidosis in Adults with Type 1 Diabetes Treated with Sotagliflozin

**DOI:** 10.1089/dia.2023.0605

**Published:** 2024-09-04

**Authors:** Schafer Boeder, Michael J. Davies, Janet B. McGill, Richard Pratley, Manon Girard, Phillip Banks, Jeremy Pettus, Satish Garg

**Affiliations:** ^1^ Division of Endocrinology and Metabolism, Department of Medicine, University of California, San Diego, La Jolla, California, USA.; ^2^ Lexicon Pharmaceuticals, Inc., The Woodlands, Texas, USA.; ^3^ Division of Endocrinology, Metabolism and Lipid Research, John T. Milliken Department of Medicine, Washington University, St. Louis, Missouri, USA.; ^4^ AdventHealth Translational Research Institute, Orlando, Florida, USA.; ^5^ Barbara Davis Center for Diabetes at the University of Colorado Denver, Aurora, Colorado, USA.

**Keywords:** Type 1 diabetes, SGLT-2/SGLT-1 inhibitors, Sotagliflozin, Diabetic ketoacidosis, Beta-hydroxybutyrate

## Abstract

**Introduction:**

Sodium glucose cotransporter inhibitors may increase beta-hydroxybutyrate (BHB) in insulin-requiring patients. We determined factors associated with BHB changes from baseline (ΔBHB) and diabetic ketoacidosis (DKA) in patients with type 1 diabetes (T1D) receiving sotagliflozin as an insulin adjunct.

**Research Design and Methods:**

This post hoc analysis compared ΔBHB levels in adults with T1D receiving sotagliflozin 400 mg or placebo for 6 months. We evaluated clinical and metabolic factors associated with ΔBHB and used logistic regression models to determine predictors associated with BHB values >0.6 and >1.5 mmol/L (inTandem3 population; *N* = 1402) or with DKA events in a pooled analysis (inTandem1–3; *N* = 2453).

**Results:**

From baseline (median, 0.13 mmol/L), median fasting BHB increased by 0.04 mmol/L (95% confidence interval, 0.03–0.05; *P* < 0.001) at 24 weeks with sotagliflozin versus placebo; 67% of patients had no or minimal changes in BHB over time. Factors associated with on-treatment BHB >0.6 or >1.5 mmol/L included baseline BHB and sotagliflozin use. Age, insulin pump use, sotagliflozin use, baseline BHB, and ΔBHB were significantly associated with DKA episodes. Independent of treatment, DKA risk increased by 18% with each 0.1-mmol/L increase in baseline BHB and by 8% with each 0.1-mmol/L increase from baseline.

**Conclusion:**

Incremental increases in baseline BHB and ΔBHB were associated with a higher DKA risk independent of treatment. Adding sotagliflozin to insulin increased median BHB over 24 weeks in patients with T1D and was associated with increased DKA events. These results highlight the importance of BHB testing and monitoring and individualizing patient education on DKA risk, mitigation, identification, and treatment.

## Introduction

Ketosis, or an increase in blood ketone levels, typically emerges in the setting of insulin insufficiency coupled with increased levels of glucagon and other counter-regulatory hormones, which leads to lipolysis and oxidation of fatty acids in the liver to ketone bodies, including beta-hydroxybutyrate (BHB) and acetoacetate.^1^ Ketoacidosis describes the presence of ketosis with concurrent anion gap metabolic acidosis (venous pH <7.3 and serum bicarbonate ≤18 mEq/L). The use of sodium glucose cotransporter (SGLT) inhibitors increases the risk of ketosis and diabetic ketoacidosis (DKA) in type 1 and type 2 diabetes (T1D and T2D, respectively).^2–9^ In patients with T1D treated with the SGLT inhibitor dapagliflozin, insulin dose reductions >20% were associated with increases in BHB as well as an increased incidence of DKA.^6^

In a pooled analysis of the year-long inTandem1 and 2 trials of the SGLT inhibitor sotagliflozin in T1D, increased DKA incidence was associated with female sex, lower baseline body mass index (BMI), use of continuous subcutaneous insulin infusion (CSII) at baseline, lower insulin requirements at baseline, and insulin dose reductions of ≥10%–20% during the trial.^5^

Recent evidence suggests that ketosis is a predictor of future DKA events in T1D.^10^ To further explore the clinical factors associated with ketosis and whether ketosis predicts DKA among adults with T1D treated with sotagliflozin as an adjunct to insulin, we evaluated changes from baseline in BHB in a post hoc analysis of the inTandem 3 trial and conducted a multivariate logistic regression analysis of pooled data from the inTandem1, 2, and 3 trials (NCT02384941, NCT02421510, and NCT02531035, respectively).^2^

## Research Design and Methods

The inTandem3 trial was a 24-week, multicenter, randomized, placebo-controlled trial of sotagliflozin added to insulin in 1402 patients with T1D. The trial was conducted in accordance with the Declaration of Helsinki, with appropriate institutional review board approvals and informed consent by patients. An independent clinical endpoint committee, blinded to treatment assignment, adjudicated DKA and other events of special interest. Trial design details and results have been published.^2^ Important exclusion criteria related to these analyses included patients with a screening BHB greater than 0.6 mmol/L or those with a history of DKA within 1 month before a screening visit, or more than 2 episodes within 6 months before the screening visit.

In this post hoc analysis, we used fasting BHB values assessed by the central laboratory at screening, baseline, and study visits at weeks 4, 8, 16, and 24. We compared median baseline and change in BHB over 24 weeks between the sotagliflozin and placebo treatment groups using a quantile regression, including treatment and the randomization stratification factors BMI (<25 or ≥25 kg/m^2^), glycated hemoglobin (A1c) (≤9% or >9%), and use of CSII at screening (yes or no).

The sotagliflozin group was divided into tertiles based on the following changes from baseline in BHB (ΔBHB) over 24 weeks: <0.01, 0.01–0.12, and ≥0.13 mmol/L. Baseline characteristics were compared across tertiles using a chi-square test of independence. Of the 699 patients originally randomized to sotagliflozin, 6 were excluded from the tertile analyses as they had no postbaseline values collected.

The relationship between change in BHB from baseline and potentially related factors (age; sex; BMI; body weight; A1C; fasting plasma glucose [FPG]; estimated glomerular filtration rate [eGFR]; urinary glucose-creatinine ratio [UGCR]; and daily basal, bolus, and total insulin doses) was examined using Spearman rank correlation for each treatment group.

The proportions of patients with at least one occurrence of a clinically important level of BHB (>0.6 or >1.5 mmol/L) during a follow-up visit were determined. The impact of each potentially related factor on the odds ratio (OR) of these BHB cut points was estimated using three different logistic regression models. The models were used to calculate OR, 95% confidence intervals (CIs), and *P*-values. The OR for a baseline covariate was calculated as the ratio of odds over one unit increase of the covariate, and it was assumed to be constant. *P*-values represent the statistical significance level of the OR differing from 1.

BHB Model 1 was adjusted for treatment, baseline A1C, baseline BHB, and age at diagnosis of T1D (<18 or ≥18 years of age). BHB Model 2 was adjusted for treatment and baseline BHB, and BHB Model 3 was adjusted for treatment, baseline BHB, and randomization stratification factors (BMI, A1C, and CSII use). The area under a receiver operating characteristic curve was calculated as a quantitative summary measure of the accuracy of prediction.

Because there were a relatively small number of DKA events in the 24-week inTandem3 trial, we pooled the inTandem3 data with those from the safety populations of the 52-week inTandem1 and 2 trials to increase the sample size in the analysis of predictors of DKA events.^2,11,12^ In all three trials, fasting BHB data were collected. The pooled data from the three studies were used in a stepwise logistic regression model to analyze associations between positively adjudicated DKA events (occurring on or after the first dose of study drug and up to 30 days after the last dose of study drug) and potential clinical risk factors among patients treated with sotagliflozin 400 mg or placebo.

In each step of the model, the incidence of study participants with at least one positively adjudicated DKA event served as the dependent variable, study number as the covariate, and risk factors, including treatment, as independent variables, with treatment duration on the natural logarithm scale as the offset. In DKA Model 1, the independent variables in the first step of the model included the following baseline variables: age at study entry; insulin delivery method (CSII vs. non-CSII); age at diagnosis of T1D (<18 or ≥18 years); duration of T1D (<20 or ≥20 years); history of DKA or ketosis; male or female sex; and baseline A1C, BMI, BHB, mean daily insulin doses (total, basal, and bolus), and urine ketones.

For DKA Model 2, treatment and change from baseline in selected measurements were used as predictor variables. The variables for the change from baseline model included body weight, BHB, percent change in daily insulin dose (total, basal, and bolus), and A1C. For patients with a DKA event, change from baseline to the last assessment before the event was used and for those without an event, change from baseline to the last study visit was used.

For both DKA models, Wald chi-square was used to determine the predictor individually. A significance level of 0.20 was required to allow a risk factor into the model, and a significance level of 0.35 was required for a risk factor to stay in the model. If the final model included a first-order interaction of a factor with treatment, then both the factor and treatment were included in the final model even if their significance level exceeded 0.35. The stepwise logistic regression model was used to obtain OR for predictors of DKA events.

## Results

**FIG. 1.**
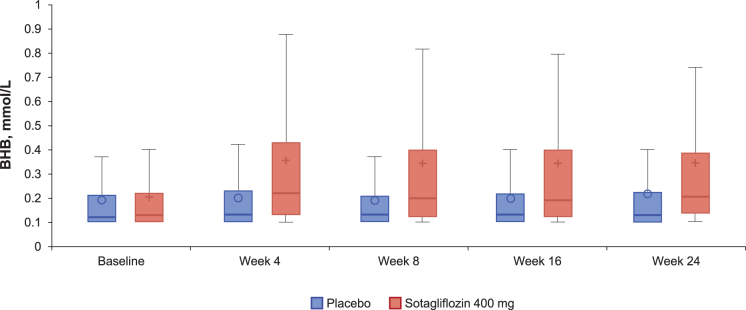
Box plot for BHB levels at each visit in patients with type 1 diabetes treated with sotagliflozin 400 mg or placebo (inTandem 3 population; *N* = 1402). Lower limit of detection is 0.1 mmol/L. Parts of the figure: line in box is the median; bottom and top of box represent the 25th and 75th percentiles, respectively; symbol (circle or plus sign) is the mean; bottom and top whiskers represent the 5th and 95th percentiles, respectively. BHB, beta-hydroxybutyrate.

Baseline median BHB (quartile 1, quartile 3 [Q1, Q3]) was 0.13 mmol/L (0.10, 0.22 mmol/L) in the sotagliflozin group and 0.12 mmol/L (0.10, 0.21 mmol/L) in the placebo group. After 24 weeks, changes in median BHB values were 0.04 mmol/L (−0.01, 0.20) and 0.00 mmol/L (−0.04, 0.05) in sotagliflozin- and placebo-treated patients, respectively (adjusted median difference = 0.04 mmol/L [95% CI, 0.03–0.05]; *P* < 0.001). Over the 24-week study period, median change in BHB was increased at week 4 in the sotagliflozin group and remained stable thereafter, while placebo-group values remained relatively unchanged over time (Fig. 1).

**FIG. 2.**
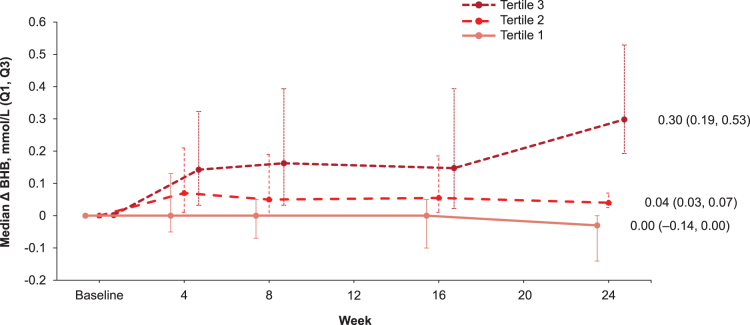
Median change from baseline in BHB in patients treated with sotagliflozin 400 mg by tertile of BHB response over 24 weeks (inTandem 3 population; *N* = 1402). Error bars represent the first and third quartile (Q1, Q3).

Patients receiving sotagliflozin who had baseline BHB values were divided into tertiles based on change in BHB from baseline to 24 weeks, including 262 patients in tertile 1 (ΔBHB <0.01 mmol/L), 200 patients in tertile 2 (ΔBHB 0.01–0.12 mmol/L), and 231 patients in tertile 3 (ΔBHB ≥0.13 mmol/L). Baseline characteristics were similar across the tertiles. Body weight and insulin doses were slightly higher in tertile 1 than in tertiles 2 or 3, but the trend was not statistically significant (Table 1). Median changes in BHB (Q1, Q3) at week 24 were −0.03 mmol/L (−0.14, 0.0 mmol/L) in tertile 1, 0.04 mmol/L (0.03, 0.07 mmol/L) in tertile 2, and 0.30 mmol/L (0.19, 0.53 mmol/L) in tertile 3. There was some overlap between tertiles at time points before week 24. Values remained stable over the 24-week trial except for a small increase in tertile 3 at week 24 (Fig. 2).

**Table 1.** Baseline Characteristics by Tertiles Based on Changes in Beta-Hydroxybutyrate over 24 Weeks in Patients with Type 1 Diabetes Receiving Sotagliflozin 400 mg (inTandem3 Population)

Characteristics	Tertile 1, BHB ≤0.0 mmol/L (*n* = 262)	Tertile 2, BHB 0.01–0.12 mmol/L (*n* = 200)	Tertile 3, BHB ≥0.13 mmol/L (*n* = 231)	*P*-value for trend
Age, years	43.5 (14.0)	42.7 (14.4)	43.6 (14.4)	0.73
Female sex	136 (51.9)	87 (43.5)	115 (49.8)	0.19
Weight, kg	83.3 (17.3)	82.5 (18.0)	81.5 (16.3)	0.52
BMI, kg/m^2^	28.7 (5.3)	28.0 (5.0)	28.1 (5.1)	0.29
<25 kg/m^2^	71 (27.1)	62 (31.0)	70 (30.3)	0.61
≥25 kg/m^2^	191 (72.9)	138 (69.0)	161 (69.7)	
Duration of diabetes, years	20.6 (12.1)	19.6 (12.0)	21.1 (13.1)	0.46
Use of CSII	101 (38.5)	85 (42.5)	86 (37.2)	0.51
Daily total insulin dose, IU/day	59.3 (29.5)	57.4 (25.6)	53.5 (26.1)	0.06
Daily basal insulin dose, IU/day	31.0 (17.7)	30.1 (15.7)	27.5 (15.0)	0.049
Daily bolus insulin dose, IU/day	28.3 (18.1)	27.3 (15.4)	26.0 (16.0)	0.31

Data are mean (SD) or *n* (%).

BHB, beta-hydroxybutyrate; BMI, body mass index; CSII, continuous subcutaneous insulin infusion; SD, standard deviation.

In both the sotagliflozin and placebo groups, small (ρ < ± 0.3) but significant correlations were found between changes in BHB and changes in FPG, body weight, bolus insulin dose, and baseline eGFR (*P* < 0.05; Table 2). Changes in UGCR and A1C were also significantly correlated with change in BHB in the sotagliflozin group (ρ ≤ 0.23; *P* < 0.05). Correlation coefficients (ρ < ± 0.15) were significant between change in BHB and change in total insulin dose and baseline A1C, and basal insulin dose in the placebo group (*P* < 0.05; Table 2).

**Table 2.** Spearman Correlation Analysis of the Change in Beta-Hydroxybutyrate at Week 24 by Potentially Related Factors (inTandem3 Population; *N* = 1402)

Characteristics	Spearman correlation (*P*-value)*^a^*	
	Placebo	Sotagliflozin 400 mg
Baseline values		
Age	0.006 (0.88)	−0.019 (0.64)
Sex	0.018 (0.65)	0.036 (0.37)
BMI	0.037 (0.36)	−0.049 (0.23)
<25 vs. ≥25 kg/m^2^	0.020 (0.62)	−0.036 (0.37)
Body weight	0.034 (0.39)	−0.031 (0.44)
eGFR	**0.081 (0.042)**	**0.081 (0.042)**
<60 vs. ≥60 mL/min/1.73 m^2^	0.020 (0.61)	0.003 (0.94)
A1C	**0.081 (0.042)**	0.007 (0.86)
FPG	**−0.132 (<0.001)**	**−0.100 (0.012)**
UGCR	0.047 (0.26)	0.018 (0.66)
Insulin dose		
Total	0.068 (0.09)	−0.056 (0.17)
Basal	**0.087 (0.029)**	−0.026 (0.52)
Bolus	0.015 (0.67)	−0.055 (0.17)
Change from baseline		
Body weight	**−0.079 (0.047)**	**−0.128 (0.001)**
A1C	−0.002 (0.97)	**0.096 (0.017)**
FPG	**0.282 (<0.001)**	**0.218 (<0.001)**
UGCR	0.027 (0.522)	**0.226 (<0.001)**
Insulin dose		
Total	**−0.110 (0.006)**	−0.047 (0.24)
Basal	−0.076 (0.06)	−0.007 (0.85)
Bolus	**−0.110 (0.006)**	**−0.106 (0.009)**

^a^ Boldface indicates statistically significant values.

A1C, glycated hemoglobin; eGFR, estimated glomerular filtration rate; FPG, fasting plasma glucose; UGCR, urine glucose creatinine ratio.

At least one BHB >0.6 mmol/L was reported in 202/672 (30.1%) patients treated with sotagliflozin and 55/678 (8.1%) patients treated with placebo, whereas at least one BHB >1.5 mmol/L was reported in 40/672 (6.0%) and 3/678 (0.4%) patients in the sotagliflozin and placebo groups, respectively. Regardless of the regression model, sotagliflozin treatment and baseline BHB levels were major predictor variables for an on-treatment BHB level >0.6 or >1.5 mmol/L being documented during the trial (Table 3). Inclusion of other factors had little or no predictive value (Table 3).

**Table 3.** Factors Predictive of Incidence of Beta-Hydroxybutyrate >0.6 or >1.5 mmol/L During the 24-Week Trial of Sotagliflozin 400 mg (inTandem3 Population; *N* = 1402)

Response term and covariate terms*^a^*	≥1 BHB >0.6 mmol/L*^b^*			≥1 BHB >1.5 mmol/L*^c^*		
	OR (95% CI),* P*-value			OR (95% CI),* P*-value		
	Model 1	Model 2	Model 3	Model 1	Model 2	Model 3
Treatment (sotagliflozin 400 mg vs **placebo**)	5.76 (4.07–8.16), <0.001	5.63 (3.99–7.95), <0.001	5.64 (4.00–7.96), <0.001	14.84 (4.50–48.94), <0.001	15.30 (4.63–50.55), <0.001	14.99 (4.56–49.29), <0.001
Baseline BHB (per 1 mmol/L increments)	35.27 (15.20–81.88), <0.001	36.38 (15.60–84.86), <0.001	35.93 (15.32–84.25), <0.001	5.66 (2.10–15.26), <0.001	5.76 (2.16–15.32), <0.001	6.06 (2.18–16.86), <0.001
Baseline A1C (per 1% increment)	0.85 (0.72–0.99), 0.046	—	—	1.21 (0.91–1.62), 0.19	—	—
Age at T1D diagnosis (≥18 vs. **<18 years**)	0.81 (0.60–1.09), 0.17	—	—	0.92 (0.49–1.72), 0.78	—	—
CSII at screening (yes vs. **no**)	—	—	0.91 (0.67–1.25), 0.57	—	—	1.31 (0.69–2.48), 0.42
Week −2 A1C (>9.0% vs. **≤9.0%**)	—	—	1.09 (0.76–1.55), 0.65	—	—	2.01 (1.04–3.91), 0.039
BMI at screening (≥25 vs. **<25 kg/m^2^**)	—	—	0.88 (0.64–1.21), 0.43	—	—	1.15 (0.58–2.315), 0.69
Area under ROC curve	0.77	0.76	0.76	0.77	0.76	0.80

^a^ Reference categories are in boldface.

^b^ No. of affected participants: sotagliflozin, 202/672 (30.1%); placebo, 55/678 (8.1%).

^c^ No. of affected participants: sotagliflozin, 40/672 (6.0%); placebo, 3/678 (0.4%).

CI, confidence interval; OR, odds ratio; ROC, receiver operating characteristics; T1D, type 1 diabetes.

As previously reported, at least one episode of positively adjudicated DKA was reported in 21 of 699 (3.0%) sotagliflozin-treated patients and 4 of 703 (0.6%) placebo-treated patients during the inTandem3 trial.^2^ In the pooled analysis of the three phase 3 inTandem trials, the sotagliflozin 400 mg arm included 1224 patients, of whom 41 (3.3%; 5.3 events per 100 patient-years) experienced a DKA event, and the placebo arm included 1229 patients, of whom 5 (0.4%; 0.6 per 100 patient-years) experienced a DKA event. In the DKA model evaluating baseline variables as predictors (Model 1), baseline BHB (per 1 mmol/L change), A1C, age, history of DKA or ketosis, and CSII use in addition to treatment with sotagliflozin 400 mg were predictive of DKA events (Table 4).

**Table 4.** Results of Stepwise Logistic Regression Model Risk Factor Analysis of Positively Adjudicated Diabetic Ketoacidosis from the Three Pooled Phase 3 inTandem Trials (Sotagliflozin 400 mg and Placebo Arms Only; *N* = 2453)

Potential risk factor*^a,b^*	Wald chi-square	*P*	OR (95% CI)
DKA Model 1 (baseline variables)			
Sotagliflozin 400 mg vs. **placebo**	21.417	<0.001	9.21 (3.60–23.58)
History of DKA or ketosis vs. **none**	10.226	0.001	4.19 (1.74–10.07)
Baseline BHB, mmol/L (per 1 mmol/L increment)	7.096	0.008	5.23 (1.55–17.67)
CSII vs. **no CSII**	6.297	0.012	2.25 (1.19–4.23)
Baseline A1C, % (per 1% increment)	5.224	0.022	1.43 (1.05–1.95)
Age at study entry, years (per 1 year increment)	4.474	0.034	0.98 (0.95–1.00)
Baseline BMI, kg/m^2^ (per 1 kg/m^2^ increment)	2.168	0.14	0.95 (0.90–1.02)
Baseline urine ketones (present or **absent**)	1.884	0.17	0.47 (0.16–1.38)
DKA Model 2 (change from baseline variables)			
Sotagliflozin 400 mg vs. **placebo**	12.395	<0.001	6.63 (2.31–19.01)
BHB, change from baseline, mmol/L (per 1 mmol/L increment)	8.680	0.003	2.11 (1.28–3.47)
Total insulin dose, percent change from baseline (per 1% increment)	3.275	0.07	0.98 (0.96–1.00)

^a^ Reference categories are in boldface.

^b^ For patients with a DKA event, change from baseline to the last assessment before the event was used in the model and for those without an event, change from baseline to the last study visit was used.

DKA, diabetic ketoacidosis.

In the DKA model evaluating change from baseline variables as predictors (Model 2), treatment with sotagliflozin and change from baseline in BHB (per 1 mmol/L change), regardless of the treatment group, were the only significant predictors. Percent change in total insulin dose was of borderline significance in the model (Table 4). When the increment of baseline BHB or change from baseline was lowered to per 0.1 mmol/L in the model, which may be more clinically relevant than the larger 1 mmol/L increments, the OR was 1.18 (95% CI, 1.04–1.33) for baseline BHB and 1.08 (95% CI, 1.03–1.13) for change from baseline in BHB.

## Discussion

In this post hoc analysis of data from the inTandem3 trial of sotagliflozin as an adjunct to insulin in patients with T1D, median BHB levels increased by 0.04 mmol/L from a baseline of 0.13 mmol/L after 24 weeks of treatment with sotagliflozin versus placebo. More than two-thirds of sotagliflozin-treated patients had a negligible increase over 24 weeks. Correlation analyses indicated that changes in body weight, UGCR, A1C, bolus insulin, and FPG were associated with change from baseline in BHB in the sotagliflozin group. Neither the baseline insulin dose nor the change in total or basal insulin doses observed in this study appeared to influence the BHB response. In a logistic regression model, sotagliflozin treatment and baseline BHB levels were consistently predictive of patients experiencing at least one clinically important BHB level of >0.6 or >1.5 mmol/L during follow-up, while baseline A1C levels also had some predictive value.

In regression models using pooled data from all three inTandem trials (to increase the robustness of the DKA predictor analysis), use of sotagliflozin 400 mg, baseline BHB, and change in BHB before a DKA event were associated with DKA events, in addition to clinical factors typically associated with increased DKA risk, such as history of DKA or ketosis, insulin pump use, baseline A1C, and age.^13,14^ As may be expected, the BHB increment used in the model increased the likelihood of a DKA event regardless of treatment. The odds of experiencing a DKA event increased by fivefold with each 1.0 mmol/L incremental increase in baseline BHB and by 18% with each 0.1 mmol/L increase.

Meanwhile, a 1.0 mmol/L increase in BHB from baseline was associated with a twofold increase in DKA risk, and a 0.1 mmol/L increase with an 8% increased risk of DKA. These results may suggest clinically useful thresholds when evaluating BHB before starting or during therapy.

These findings have important clinical implications. First, they are consistent with a recent post hoc analysis of data from the Empagliflozin as Adjunctive to Insulin Therapy (EASE)-2 and -3 trials of empagliflozin in T1D, which showed a significant association between DKA events and preceding BHB increases in 484 patients who received placebo during the trial. Moreover, the EASE investigators found that BHB levels ≥0.8 mmol/L nearly tripled the 6- to 12-month risk of DKA in this population.^10^ This is generally consistent with the aforementioned fivefold increase in the likelihood of experiencing a DKA event with every 1.0 mmol/L increase in baseline BHB in the present analysis.

In clinical trials in patients with T1D, the incidence of DKA was similar across SGLT2 inhibitors, with some evidence of higher incidence of DKA with increasing dose.^3–5,7,8,15,16^ Thus, the American Diabetes Association recommends stopping or delaying SGLT inhibitors in cases of ketonemia (defined by elevated BHB).^17^ Consistent with this recommendation and based on the results presented here, we suggest checking BHB levels in all insulin-using patients before initiating an SGLT inhibitor. If BHB is elevated, the SGLT inhibitor should not be started until BHB levels have normalized. Our findings also support the recommendations of an international consensus report, which, in the absence of data supporting specific testing regimens, suggested individualizing BHB testing frequency in patients with T1D using SGLT inhibitors based on lifestyle and risk factors.

The consensus group also recommended monitoring BHB in all patients with potential DKA symptoms, changes to diet or insulin, or acute events (e.g., pump failure, illness, or other physiological stress).^3^ Our results further suggest that BHB should be monitored to guide treatment decisions in these settings regardless of the use of SGLT inhibitors. These measures should be considered in addition to standard-of-care education for patients about DKA risk and mitigation. During the inTandem program, implementation of a DKA risk management strategy was associated with a reduction in DKA incidence.^5,18^

As described in the international consensus report, patients with BHB levels between 0.6 and 1.5 mmol/L should consume carbohydrates and fluids, with appropriate rapid-acting insulin and frequent checks of blood or urine ketones and blood glucose until elevated BHB levels resolve. A BHB level ≥1.6 (i.e., >1.5) mmol/L is a sign of “impending DKA” and may necessitate medical attention, while a BHB >3.0 mmol/L calls for immediate medical attention for probable DKA.^3^

For this post hoc analysis, we used fasting BHB collected at clinical visits over a 24-week period in a large cohort of patients. However, during the trial, BHB values were not systematically captured during at-home assessments, which may have provided more insights to BHB changes. Factors not measured in the present trial (e.g., glucagon, C-peptide) may have also played a role in the BHB responses or been predictors of DKA events.

## Conclusions

Sotagliflozin as an adjunct to insulin led to small but statistically significant increases in BHB over 24 weeks. In fact, in approximately two-thirds of patients, sotagliflozin did not lead to any appreciable increase in BHB. Baseline BHB, changes in BHB, and sotagliflozin use were the main predictors associated with clinically meaningful BHB elevations or DKA events. Common predictors of DKA risk, including age, gender, insulin pump use, and A1C, were also observed in the analyses. These results highlight the importance of patient selection based on BHB testing before starting SGLT inhibitors and education regarding risk, mitigation, identification, and treatment strategies for ketosis and DKA. Our findings further suggest that BHB should be monitored in all patients with T1D, regardless of SGLT inhibitor use, although additional research is needed to determine the optimal frequency of BHB monitoring.

## References

[1] Kitabchi AE, Umpierrez GE, Miles JM, et al. Hyperglycemic crises in adult patients with diabetes. Diabetes Care 2009;32(7):1335–1343; doi: 10.2337/dc09-9032 19564476 PMC2699725

[2] Garg SK, Henry RR, Banks P, et al. Effects of sotagliflozin added to insulin in patients with type 1 diabetes. N Engl J Med 2017;377(2337–2348; doi: 10.1056/NEJMoa1708337 28899222

[3] Danne T, Garg S, Peters AL, et al. International consensus on risk management of diabetic ketoacidosis in patients with type 1 diabetes treated with sodium-glucose cotransporter (SGLT) inhibitors. Diabetes Care 2019;42(6):1147–1154; doi: 10.2337/dc18-2316 30728224 PMC6973545

[4] Peters AL, Buschur EO, Buse JB, et al. Euglycemic diabetic ketoacidosis: A potential complication of treatment with sodium-glucose cotransporter 2 inhibition. Diabetes Care 2015;38(9):1687–1693; doi: 10.2337/dc15-0843 26078479 PMC4542270

[5] Peters AL, McGuire DK, Danne T, et al. Diabetic ketoacidosis and related events with sotagliflozin added to insulin in adults with type 1 diabetes: A pooled analysis of the inTandem 1 and 2 studies. Diabetes Care 2020;43(11):2713–2720; doi: 10.2337/dc20-0924 32928957 PMC7576419

[6] Henry RR, Dandona P, Pettus J, et al. Dapagliflozin in patients with type 1 diabetes: A post hoc analysis of the effect of insulin dose adjustments on 24-hour continuously monitored mean glucose and fasting beta-hydroxybutyrate levels in a phase IIa pilot study. Diabetes Obes Metab 2017;19(6):814–821; doi: 10.1111/dom.12882 28098426

[7] Peters AL, Henry RR, Thakkar P, et al. Diabetic ketoacidosis with canagliflozin, a sodium-glucose cotransporter 2 inhibitor, in patients with type 1 diabetes. Diabetes Care 2016;39(4):532–538; doi: 10.2337/dc15-1995 26989182

[8] Rosenstock J, Marquard J, Laffel LM, et al. Empagliflozin as adjunctive to insulin therapy in type 1 diabetes: The EASE trials. Diabetes Care 2018;41(12):2560–2569; doi: 10.2337/dc18-1749 30287422

[9] Marilly E, Cottin J, Cabrera N, et al. SGLT2 inhibitors in type 2 diabetes: A systematic review and meta-analysis of cardiovascular outcome trials balancing their risks and benefits. Diabetologia 2022;65(12):2000–2010; doi: 10.1007/s00125-022-05773-8 35925319

[10] Song C, Dhaliwal S, Bapat P, et al. Point-of-care capillary blood ketone measurements and the prediction of future ketoacidosis risk in type 1 diabetes. Diabetes Care 2023;46(11):1973–1977; doi: 10.2337/dc23-0840 37616393

[11] Buse JB, Garg SK, Rosenstock J, et al. Sotagliflozin in combination with optimized insulin therapy in adults with type 1 diabetes: The North American inTandem1 study. Diabetes Care 2018;41(9):1970–1980; doi: 10.2337/dc18-0343 29937430 PMC6105319

[12] Danne T, Cariou B, Banks P, et al. HbA1c and hypoglycemia reduction at 24 and 52 weeks with sotagliflozin in combination with insulin in adults with type 1 diabetes: The European inTandem2 study. Diabetes Care 2018;41(9):1981–1990; doi: 10.2337/dc18-2316 29937431

[13] Foster NC, Beck RW, Miller KM, et al. State of type 1 diabetes management and outcomes from the T1D Exchange in 2016–2018. Diabetes Technol Ther 2019;21(2):66–72; doi: 10.1089/dia.2018.0384 30657336 PMC7061293

[14] Pettus JH, Zhou FL, Shepherd L, et al. Incidences of severe hypoglycemia and diabetic ketoacidosis and prevalence of microvascular complications stratified by age and glycemic control in U.S. adult patients with type 1 diabetes: A real-world study. Diabetes Care 2019;42(12):2220–2227; doi: 10.2337/dc19-0830 31548241

[15] Taylor SI, Blau JE, Rother KI, et al. SGLT2 inhibitors as adjunctive therapy for type 1 diabetes: Balancing benefits and risks. Lancet Diabetes Endocrinol 2019;7(12):949–958; doi: 10.1016/s2213-8587(19)30154-8 31585721 PMC6872914

[16] Mathieu C, Dandona P, Gillard P, et al. Efficacy and safety of dapagliflozin in patients with inadequately controlled type 1 diabetes (the DEPICT-2 Study): 24-Week results from a randomized controlled trial. Diabetes Care 2018;41(9):1938–1946; doi: 10.2337/dc18-0623 30026335

[17] American Diabetes Association. 16. Diabetes care in the hospital: Standards of care in diabetes—2024. Diabetes Care 2024;47(Suppl. 1):S295–S306; doi: 10.2337/dc24-S016 38078585 PMC10725815

[18] Garg SK, Peters AL, Buse JB, et al. Strategy for mitigating DKA risk in patients with type 1 diabetes on adjunctive treatment with SGLT Inhibitors: A STICH protocol. Diabetes Technol Ther 2018;20(9):571–575; doi: 10.1089/dia.2018.0246 30129772

